# Polarimetric Calibration and Quality Assessment of the GF-3 Satellite Images

**DOI:** 10.3390/s18020403

**Published:** 2018-01-30

**Authors:** Yonglei Chang, Pingxiang Li, Jie Yang, Jinqi Zhao, Lingli Zhao, Lei Shi

**Affiliations:** 1 State Key Laboratory of Information Engineering in Surveying, Mapping and Remote Sensing, Wuhan University, Wuhan 430079, China; changyonglei@whu.edu.cn (Y.C.); yangj@whu.edu.cn (J.Y.); masurq@whu.edu.cn (J.Z.); shi.lei@whu.edu.cn (L.S.); 2 School of Remote Sensing and Engineering, Wuhan University, Wuhan 430079, China; zhaolingli@whu.edu.cn

**Keywords:** polarimetric calibration, PolSAR, GaoFen-3, image quality, reflection symmetry

## Abstract

The GaoFen-3 (GF-3) satellite is the first fully polarimetric synthetic aperture radar (SAR) satellite designed for civil use in China. The satellite operates in the C-band and has 12 imaging modes for various applications. Three fully polarimetric SAR (PolSAR) imaging modes are provided with a resolution of up to 8 m. Although polarimetric calibration (PolCAL) of the SAR system is periodically undertaken, there is still some residual distortion in the images. In order to assess the polarimetric accuracy of this satellite and improve the image quality, we analyzed the polarimetric distortion errors and performed a PolCAL experiment based on scattering properties and corner reflectors. The experiment indicates that the GF-3 images can meet the satellite’s polarimetric accuracy requirements, i.e., a channel imbalance of 0.5 dB in amplitude and ±10 degrees in phase and a crosstalk accuracy of −35 dB. However, some images still contain residual polarimetric distortion. The experiment also shows that the residual errors of the GF-3 standard images can be diminished after further PolCAL, with a channel imbalance of 0.26 dB in amplitude and ±0.2 degrees in phase and a crosstalk accuracy of −42 dB.

## 1. Introduction

The GaoFen-3 (GF-3) satellite, which operates in the C-band, is the first fully polarimetric synthetic aperture radar (PolSAR) imaging satellite in China [1]. It was launched on 10 August 2016 and has a design life of eight years. The satellite has 12 imaging modes, the most of any SAR satellite in the world. The imaging modes include StripMap mode, ScanSAR mode, Spotlight mode, wave imaging mode, and ultra-fine strip mode [2]. The details of the different imaging modes are provided in Table 1. GF-3 can achieve an image width of 650 km at a resolution of 500 m and a 10-km image width at a resolution of 1 m [3]. Therefore, the satellite can serve many industries and research applications, such as disaster monitoring, water conservancy, meteorological observation, marine monitoring, and global environmental studies. Furthermore, the system also has three fully polarimetric SAR imaging modes, making it efficient in geographical interpretation and quantitative research [4].

A PolSAR system measures the scattering information of the Earth’s surface with different polarimetric microwaves. Generally, it transmits vertical polarization wave [5] and receives echoes in vertical and horizontal polarizations simultaneously. This is followed by transmitting a horizontal polarization wave and, again, receiving echoes in vertical and horizontal polarizations simultaneously. In this way, the system obtains four images of different polarization channel in one area. Generally speaking, PolSAR technology discriminates ground objects by the coherence and difference of these channel images. Therefore, before PolSAR images can be used for further research or applications, polarimetric calibration (PolCAL) is needed to ensure the relative amplitude and phase of the different image channels [5,6]. The polarization accuracy is very important for PolSAR satellites such as Radarsat-2, Alos-2, TerraSAR-X, and COSMO-SkyMed. The crosstalk accuracy of Radarsat-2 is −30 dB and the channel imbalance is 0.5 dB in amplitude and 5 degrees in phase [7]. The accuracy requirement of Alos-2 is −30 dB in crosstalk and 0.4 dB in channel imbalance amplitude and 5 degrees in channel imbalance phase, and the practical accuracy in some test sites is much better [8] . After the launch of the GF-3 satellite, a series of ground calibration experiments were carried out in Etuoke Banner, Inner Mongolia. Furthermore, the data processing system was improved in March 2017. It is therefore necessary to again assess the polarimetric accuracy of the satellite images.

The term “PolCAL” in SAR has two meanings. The first is to determine the polarimetric distortion matrices (PDM) of a PolSAR system, which express the polarimetric transformation between transmission and reception. The other is to convert uncalibrated PolSAR data to calibrated data using these PDMs [9]. A lot of research has been done on PolCAL since the first PolSAR system, i.e., Airborne Synthetic Aperture Radar (AIRSAR), was developed [10]. The early PolCAL methods were designed using different kinds of corner reflectors (CRs) or polarimetric active radar calibrators (PARCs) [11]. Subsequently, some particular scattering targets such as bare soil, desert, and grassland have been investigated and taken as PolCAL references [12,13,14]. Rainforest has also been studied for PolCAL in some research [9,15]. The current PolCAL schemes for PolSAR systems usually use both distributed targets and CRs. Although periodical PolCAL has been performed on the GF-3 system, there is still some channel distortion in the distributed images. In order to assess the polarimetric accuracy of the GF-3 satellite and improve the image quality, we analyzed the polarimetric errors and performed PolCAL based on scattering symmetry and ground CRs.

In this paper, the polarimetric errors of the GF-3 system are described in Section 2. Section 3 describes the PolCAL method and the accuracy verification method used in this research. The experiments and results are discussed in Section 4, and the conclusions are presented in Section 5.

## 2. Polarimetric Error Analysis for the GF-3 Satellite

Generally speaking, the data observed by a PolSAR system is in the format of scattering matrix, which is a 2 × 2 complex matrix that characterizes the response from one target. However, during the observation, the real scattering matrix is affected by polarimetric distortion. The distortion is made up of the crosstalk of the different channels, the amplitude and phase imbalance between the co-polarization (co-pol) channels (co-imba), the imbalance between the two cross-polarization (cross-pol) channels (cross-imba), the system noise, and the Faraday rotation. The relationship between the scattering matrix and the polarimetric distortion can be represented by the PolSAR distortion model [16]:

(1) $$\begin{array}{c} \left(\begin{array}{cc} O_{hh} & O_{hv}\\ O_{vh} & O_{vv} \end{array}\right)=A\left[\begin{array}{cc} 1 & w\\ u & 1 \end{array}\right]\left[\begin{array}{cc} k & 0\\ 0 & 1 \end{array}\right]\left[\begin{array}{cc} \cos\Omega & \sin\Omega \\ -\sin\Omega & \cos\Omega \end{array}\right]\left(\begin{array}{cc} S_{hh} & S_{hv}\\ S_{vh} & S_{vv} \end{array}\right)*\\ \;[\begin{array}{cc} \cos\Omega & \sin\Omega \\ -\sin\Omega & \cos\Omega \end{array}]\left[\begin{array}{cc} k & 0\\ 0 & 1 \end{array}\right]\left[\begin{array}{cc} \alpha & 0\\ 0 & 1 \end{array}\right]\left[\begin{array}{cc} 1 & z\\ v & 1 \end{array}\right]+\left[\begin{array}{cc} N_{hh} & N_{hv}\\ N_{vh} & N_{vv} \end{array}\right] \end{array}.$$

In the model, $S_{hh},S_{hv},S_{vh},S_{vv}$ compose the true scattering matrix of the ground object, with each of them representing a polarization channel. $O_{hh},O_{hv},O_{vh},O_{vv}$ compose the observation matrix gained by the PolSAR system. The u,w,v,z parameters are the crosstalk distortions. The k term is the co-pol channel imbalance error, and α is the cross-pol channel imbalance error. Argument A is the absolute calibration factor that will be used in the radiometric calibration. Parameter Ω represents the Faraday rotation angle. $N_{hh},N_{hv},N_{vh},N_{vv}$ represent the system noise of the different channels.

### 2.1. Crosstalk Error

Currently, spaceborne PolSAR systems use transmit-receive modules (TRMs) to emit or detect the microwave signals. The system alternately transmits horizontal or vertical polarization microwaves, and then receives horizontal and vertical polarization backscattering waves simultaneously but separately with different modules. In this operation, however, the polarimetric characteristics of the processing signals are not always pure. In other words, the horizontal polarization signal is often mixed with some amount of vertical signal, or vice versa, both in transmitting and receiving, which is termed the crosstalk. The sources of crosstalk are mostly the TRMs and antenna systems [17].

The crosstalk error increases the magnitude of the cross-pol images relatively, and the target will be more likely to display volume scattering properties. Most PolSAR applications require that the crosstalk should be less than −32 dB [18]. Meanwhile, for geophysical parameter inversion research and multitemporal studies over large areas, a higher accuracy is needed, and the crosstalk should be less than −35 dB [19,20,21].

### 2.2. Channel Imbalance Error

Generally speaking, PolSAR technology discriminates ground objects by the coherence and difference of the four polarization channels. To guarantee that the coherence or difference only derives from the object’s signature, the PolSAR systems should transmit or receive the polarimetric signals with the same antenna gain. The channel imbalance results from the antenna gain error, and it describes the amplitude or phase unconformity of the different polarization channels, both in transmitting and receiving [22].

According to the influence on the images, the channel imbalance can be divided into two parts, i.e., the co-polarization channel imbalance (co-imba) and the cross-polarization channel imbalance (cross-imba). The co-imba mainly causes the distortion between the co-pol channels, i.e., the HH (transmitting in horizontal polarization and receiving in horizontal polarization) and VV (transmitting in vertical polarization and receiving in vertical polarization) polarizations. The cross-imba mainly induces distortion between HV (transmitting in vertical polarization and receiving in horizontal polarization) and VH (transmitting in horizontal polarization and receiving in vertical polarization) polarizations, deforming the scattering reciprocity.

### 2.3. Faraday Rotation Error

When an electromagnetic wave propagates through an external magnetic field, the polarization plane rotates around the radar line of sight with the Faraday rotation angle (FRA). Considering the ionosphere layer and the geomagnetic field, the FRA is always considered for spaceborne SAR systems. The FRA is determined mostly by the transmission frequency, and it is also proportional to the total electron content (TEC) resulting from solar activity, the angle between the line of sight and geomagnetic, and also the geomagnetic flux density. The theoretical value is given by:

(2) $$\Omega =\frac{K}{f^{2}}\langle B\cdot \cos\varphi \cdot \sec\theta_{0}\cdot TEC\rangle ,$$

where $K=2.365\times 10^{4}$ in SI units, and is the transmission frequency (in Hz). TEC stands for the electrons per square meter, and parameter B is the geomagnetic flux density (in tesla). Term φ is the angle between the geomagnetic field vector and the radar line of sight (in radians), and $\theta_{0}$ is the incidence angle. The symbol 〈·〉 indicates an averaging operation.

The FRA can be estimated from the PolSAR data by the scattering reciprocity [23], but other polarimetric distortions may reduce the confidence. Thus, methods with external calibrators can calculate this parameter more accurately [24]. According to the current research, the FRA is apparent in low-frequency PolSAR systems such as the L- and P-band systems, but is not an issue for the C- or X-band satellites [9]. As GF-3 operates in the C-band, we do not discuss the FRA any further.

### 2.4. Random System Noise Error

System noise can arise from any part of the SAR system. It can result from electronic leakage or the physical temperature of the equipment. In most observation cases, the system noise is regarded as thermal noise, and obeys a Gaussian distribution of white noise characteristics. Therefore, the system noise is additive in PolSAR images, and is independent of the signal polarization. Using the scattering reciprocity hypothesis, the noise can be represented in the form of the signal-to-noise ratio (SNR):

(3) $$SNR=10\cdot \log_{10}\left(\frac{\left|C_{22}\right|-\left|C_{23}\right|}{1/4\cdot \left(C_{11}+C_{22}+C_{33}+C_{44}\right)}\right),$$

where $C_{*}$ is the covariance matrix element transformed from the scattering matrix [25]. As the system noise can disturb the estimation accuracy of the other polarimetric distortions, it should be considered in PolCAL, although it cannot be eliminated, due to its random property.

### 2.5. QualifyValue Adjustment

The standard GF-3 multi-polarization images must be carried on QualifyValue adjustment before use. During the imaging processing, the numerical range of the observed data is rectified. However, the different channel images are rectified with different parameters, which will change the polarimetric properties. Furthermore, every image has different rectifying parameters. Therefore, before we proceed with any treatment of the GF-3 images, we need to check the QualifyValue parameters for each channel in the metadata, and undertake QualifyValue adjustment using the following formula:

(4) $$DN_{true}=DN_{\mathrm{image}}*QualifyValue/32767,$$

where the $DN_{\mathrm{image}}$ term stands for the data in one polarization channel of GF-3 standard images. Parameter $DN_{true}$ is the true data we want to process for further research. QualifyValue is the scale parameter saved in the metadata, which is different for every polarization channel.

## 3. Calibration Method and Processing

Reflection symmetry and scattering reciprocity targets are distributed widely on the Earth’s surface, and they also have some particular characteristics in mathematical expression. Accordingly, they are commonly explored in PolCAL, and are still utilized in calibration of the current PolSAR systems and PolCAL algorithm research. Considering this, we performed the PolCAL and accuracy assessment based on reflection symmetry and scattering reciprocity. Meanwhile, to assess the images more objectively, ground CRs were also used.

### 3.1. PolCAL Method Based on Reflection Symmetry

To suppress the randomness of the PolCAL errors and obtain stable signatures for the PolCAL reference targets, the calculation of crosstalk and cross-imba is carried out on the average covariance matrix of the image. It should also be noted that the distortion errors generally vary with the image range. Considering these factors, we divide the measured image into different strips along the image range, and generate the average covariance matrix. On the other hand, since the reflection symmetry property can provide stable constraints for PolCAL, it is reasonable to extract reference samples that satisfy reflection symmetry before calibration. There are some studies that focus on PolCAL samples extraction, and here we extract these samples by using helix scattering. The pixels with low helix scattering component will be extracted as PolCAL samples [10,26]. From the PolSAR distortion model in Equation (1), we obtain the vector form, and then gain the average covariance matrix [27]:

(5) $$\begin{array}{l} \left[\begin{array}{c} O_{hh}\\ O_{hv}\\ O_{vh}\\ O_{vv} \end{array}\right]=A\left[\begin{array}{cccc} 1 & v & w & wv\\ z & 1 & wz & w\\ u & uv & 1 & v\\ uz & u & z & 1 \end{array}\right]\left[\begin{array}{cccc} \alpha & 0 & 0 & 0\\ 0 & \alpha^{-1} & 0 & 0\\ 0 & 0 & \alpha & 0\\ 0 & 0 & 0 & \alpha^{-1} \end{array}\right]\left[\begin{array}{cccc} k & 0 & 0 & 0\\ 0 & 1 & 0 & 0\\ 0 & 0 & 1 & 0\\ 0 & 0 & 0 & k^{-1} \end{array}\right]\left[\begin{array}{c} S_{hh}\\ S_{hv}\\ S_{vh}\\ S_{vv} \end{array}\right]+\left[\begin{array}{c} N_{hh}\\ N_{hv}\\ N_{vh}\\ N_{vv} \end{array}\right]\\ \Rightarrow \left[O\right]=A\left[P\right]\left[F\right]\left[K\right]\left[S\right]+\left[N\right] \end{array}$$

(6) $$\left[M\right]=\langle \left[\begin{array}{c} O_{hh}\\ O_{hv}\\ O_{vh}\\ O_{vv} \end{array}\right]\cdot \left[\begin{array}{cccc} O_{hh}^{*} & O_{hv}^{*} & O_{vh}^{*} & O_{vv}^{*} \end{array}\right]\rangle ,$$

where matrix *P* is the crosstalk distortion matrix, and matrix *F* is the cross-imba matrix. Matrix *K* represents the co-imba. These three matrices are also referred to as PDMs. The superscript * means the complex conjugate operation, and matrix *M* is a 4 × 4 complex matrix that represents the measured image.

The scattering mechanism of reflection symmetry mainly displayed by distributed targets such as grassland, desert, public squares, and bare soil. This means that the objects that satisfy this mechanism are symmetric by the line of sight in radiometric scattering. Theoretically, the covariance matrix of these targets is [25]:

(7) $$\left[C\right]=\left(\begin{array}{ccc} \langle S_{hh}S_{hh}^{*}\rangle & 0 & \langle S_{hh}S_{vv}^{*}\rangle \\ 0 & \langle S_{hv}S_{hv}^{*}\rangle & 0\\ \langle S_{vv}S_{hh}^{*}\rangle & 0 & \langle S_{vv}S_{vv}^{*}\rangle \end{array}\right),$$

where the symbol 〈·〉 indicates an averaging operation. The formula shows that the co-pol and cross-pol have zero coherence. Using the zero elements in the covariance matrix, and substituting them into the PolCAL distortion model, the PDMs can be calculated with the following formulas [27]:

(8) $$\begin{array}{l} u=(M_{44}M_{21}-M_{41}M_{24})/(M_{44}M_{11}-M_{41}M_{14})\\ v=(M_{11}M_{24}-M_{21}M_{14})/(M_{44}M_{11}-M_{41}M_{14})\\ z=(M_{44}M_{31}-M_{41}M_{34})/(M_{44}M_{11}-M_{41}M_{14})\\ w=(M_{11}M_{34}-M_{31}M_{14})/(M_{44}M_{11}-M_{41}M_{14}), \end{array}$$

where $M_{*}$ is the element of the average covariance matrix M. Accordingly, the cross-imba is calculated with [5]:

(9) $$\alpha ={\left(\frac{M_{32}-u^{*}(zM_{11}+wM_{41})-v^{*}(zM_{14}+wM_{44})}{M_{33}-z^{*}(zM_{11}+wM_{41})-w^{*}(zM_{14}+wM_{44})}\right)}^{*}.$$

Although there are some algorithms that calculate the co-imba by the use of natural targets such as bare soil [13], the prevailing and stable method relies on manmade calibrators. PARCs have a better signal-to-clutter ratio, but they are expensive. Triangular CRs are often used in PolCAL, and the parameter is calculated as:

(10) $$\left|k\right|={\left|\frac{O_{hh}O_{hh}^{*}}{O_{vv}O_{vv}^{*}}\right|}^{\frac{1}{4}}\;,\;\arg\left(k\right)=\frac{1}{2}\arg\left(O_{hh}O_{vv}^{*}\right).$$

### 3.2. PolCAL Method Based on Scattering Reciprocity

Apart from some particular manmade targets, most scattering media conform to the reciprocity property, i.e., $S_{hv}=S_{vh}$, which leads to the following form of covariance matrix [16]:

(11) $$\left[C\right]=\left[\begin{array}{cccc} C_{hhhh} & A^{*} & A^{*} & C_{hhvv}\\ A & \beta & \beta^{\prime } & B\\ A & \beta^{\prime } & \beta & B\\ C_{vvhh} & B^{*} & B^{*} & C_{vvvv} \end{array}\right],$$

in which the superscript * is the complex conjugate operator, the values of β and $\beta^{\prime }$ are real, and the others are complex. *A* and *B* are the complex coherence between the co-pol and cross-pol channels. Since reciprocity implies that the two cross-pol channels are identical, for calibrated data, all of their correlations must in fact be identical.

The PolCAL algorithm based on scattering reciprocity is an iterative process, and is widely used in the present PolSAR systems. We only give the calculation of the polarimetric distortion parameters here, and more details can be found in [16].

This amplitude of cross-imba is calculated by the power of the two cross-polarizations, and the phase is calculated by their coherence:

(12) $$\alpha ={\left(\langle M_{vhvh}\rangle /\langle M_{hvhv}\rangle \right)}^{1/4}\exp\left(i*Angle\left(\langle M_{vhhv}\rangle \right)/2\right).$$

In the equation, the symbol Angle(·) is the operator that calculates the phase of parameters. Subsequently, the calculated error is removed to generate the updated covariance matrix by matrix multiplication $\left[M^{\prime }\right]={\left[F\right]}_{}^{{}^{-1}}\left[M\right]{\left[F\right]}_{}^{{}^{-H}}$.

In every iteration, the crosstalk is calculated by the coherence of the cross-pol and co-pol channels, and the computation is based on the updated covariance matrix:

(13) $$\begin{array}{l} \left(M_{vhhh}^{\prime }+M_{hvhh}^{\prime }\right)/2≅\left(M_{vhhh}^{\prime }-\Delta uM_{hhhh}^{\prime }-\Delta vM_{vvhh}^{\prime }-\Delta v_{}^{*}M_{vhhv}^{\prime }-\Delta w_{}^{*}M_{vhvh}^{\prime }\right)\\ \left(M_{vhhh}^{\prime }+M_{hvhh}^{\prime }\right)/2≅\left(M_{hvhh}^{\prime }-\Delta w_{}M_{vvhh}^{\prime }-\Delta z_{}M_{hhhh}^{\prime }-\Delta v_{}^{*}M_{hvhv}^{\prime }-\Delta w_{}^{*}M_{hvvh}^{\prime }\right)\\ \left(M_{vhvv}^{\prime }+M_{hvvv}^{\prime }\right)/2≅\left(M_{vhvv}^{\prime }-\Delta u_{}M_{hhvv}^{\prime }-\Delta v_{}M_{vvvv}^{\prime }-\Delta u_{}^{*}M_{vhhv}^{\prime }-\Delta z_{}^{*}M_{vhvh}^{\prime }\right)\\ \left(M_{vhvv}^{\prime }+M_{hvvv}^{\prime }\right)/2≅\left(M_{hvvv}^{\prime }-\Delta w_{}M_{vvvv}^{\prime }-\Delta z_{}M_{hhvv}^{\prime }-\Delta u_{}^{*}M_{hvhv}^{\prime }-\Delta z_{}^{*}M_{hvvh}^{\prime }\right) \end{array}.$$

In the formula, Δu,Δv,Δw,Δz are the corrections of the crosstalk, and the update of the crosstalk can be depicted as:

(14) $${\left[u,v,w,z\right]}_{}^{T}={\left[u,v,w,z\right]}_{}^{T}+{\left[\Delta u_{},\Delta v_{},\Delta w_{},\Delta z_{}\right]}^{T}.$$

The calculations of the co-imba error and system noise are independent of the previous calculation, and their estimation is carried out in the same way as the reflection symmetry method.

## 4. Experiments

### 4.1. Experimental Data

In this study, GF-3 satellite images of the full-polarimetric Strip I mode were used as the experimental data. The resolution of this imagery is 8 m, and they are single look complex (SLC) images. To assess the image quality and verify the PolCAL improvement more objectively, 14 images from the two experimental sites were tested. The specific information is shown in Table 2.

The first experimental area was Etuoke Banner of Inner Mongolia. Most of the land cover of this area is sparse grassland, which is perfect for PolCAL, and there are also some salt lakes, towns, farmland, and hillsides. In order to evaluate the stability of the satellite system, researchers from the Institute of Electronics, Chinese Academy of Sciences (IECAS), set up CRs and PARCs in this area in June and July 2017, obtaining valuable ground data. The city of Wuhan in Hubei province was also selected as an experimental area, and the land cover of this area mainly are buildings, water area, forests, and farmland.

### 4.2. Experimental Design

For the Etuoke Banner site, there were two images of the same orbit each day. One of the images had ground CRs, so we took this image to calculate the PDMs. We then calibrated both of the images with the same PDMs. We used the reflection symmetry method to PolCAL the images, and used both methods to assess the accuracy.

For the Wuhan site, there was only one image each day, and there was no manmade CR in the images. Generally, the methods described in this paper use distributed targets that satisfy certain assumptions to calculate crosstalk and cross-imba PDMs, and the methods use at least one manmade CR to estimate co-imba PDM. Therefore, we only evaluated the crosstalk and the cross-imba here. We calculated these two polarimetric errors in each image firstly to see the image quality, and then calibrated the images with the errors that were calculated by the same image.

### 4.3. Experimental Results and Analysis

Since the PDMs vary with range, we first divided the measured images into different strips along the image range. In this experiment, the size of the strips was 100*Ncol pixels, where the term ‘Ncol’ means the size of the image in the azimuth direction. Secondly, we utilized an automatic extracting method to extract proper reference samples for PolCAL [10], and we then computed the average covariance matrices. After the PolCAL, we carried out the accuracy analysis on both the calibrated and uncalibrated images.

**Figure 1:**
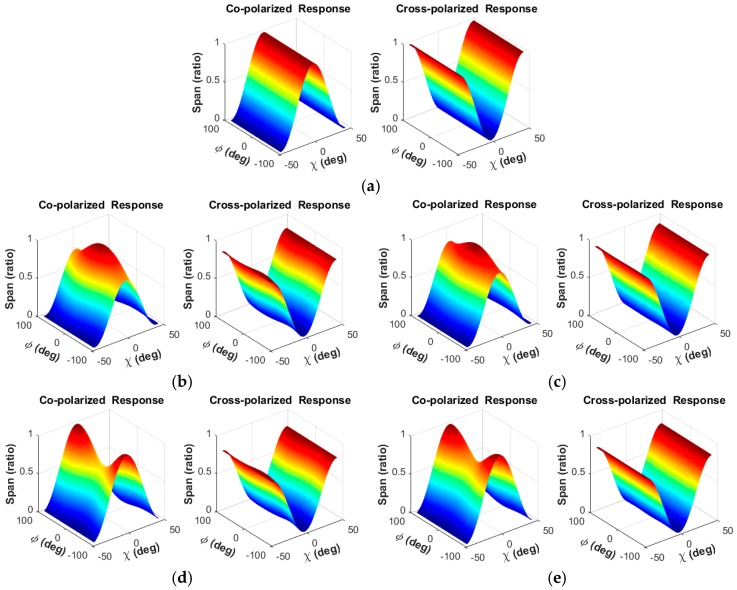

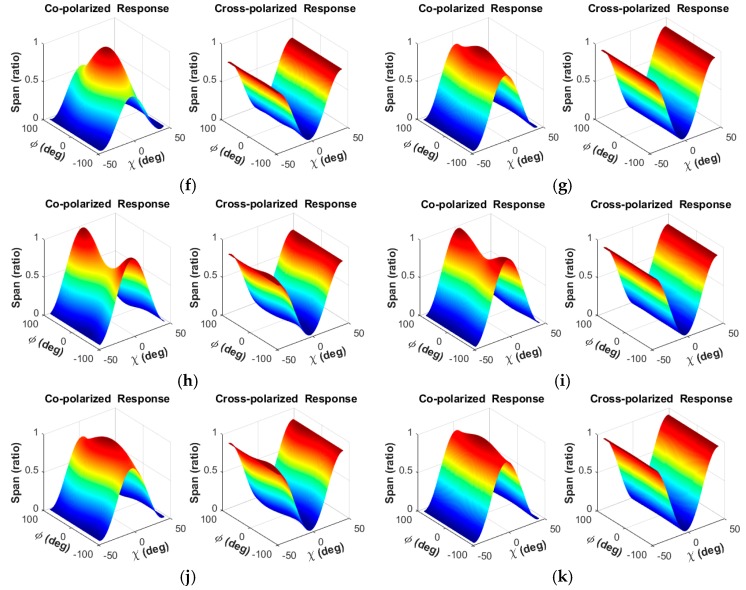
The co-pol and cross-pol response signature of CRs. The signatures show the normalized synthetic span value at different polarization pattern (expressed in a combination of ellipse orientation angel *φ* and ellipticity angle *χ*). These charts are: (**a**) theoretical PRS form of a triangular CR; (**b**,**c**) the PRSs before PolCAL and after PolCAL of image 0002441640; (**d**,**e**) the PRSs before PolCAL and after PolCAL of image 0002441191; (**f**,**g**) the PRSs before PolCAL and after PolCAL of image 0002463755; (**h**,**i**) the PRSs before PolCAL and after PolCAL of image 0002475549; (**j**,**k**) the PRSs before PolCAL and after PolCAL of image 0002491702. There are two PRS pictures for each image: the left one is the PRSs before calibration, and the right one is the PRSs after calibration.

The polarization response signatures (PRS) of the CRs in the Etuoke Banner site are shown in Figure 1. There were more than five CRs in each image, and all of them were trihedral angle reflectors. We used three CRs to estimate the PDMs and one for verification. The PRS is widely used in PolCAL, and its main purpose is to check the correctness of the calibration. The PRS shows the normalized synthetic span value at different polarization patterns, which are expressed in a combination of ellipse orientation angel *φ* (degree) and ellipticity angle *χ* (degree). The co-polarized response is synthesized when the polarization of emission wave and receiving wave are the same, and the cross-polarized response is synthesized when they are orthogonal [28]. 

The first PRS picture is the theoretical form of a triangular CR. There are two PRS pictures for each image: the left one is the PRSs before calibration, and the right one is the PRSs after calibration. The uncalibrated pictures show that there is still some polarimetric distortion in the images after the periodic systematic calibration. Secondly, some of the PRSs are much closer to the theoretical ones, which indicates that the periodic calibration is useful. On the other hand, the calibrated PRSs appear more closely to the theoretical form. After PolCAL, the peak lines of co-polarized PRSs fluctuate less, and the bottom lines of cross-polarized PRSs become straighter. This means that the further PolCAL processing can suppress the residual error and improve the polarimetric quality.

The quantitative analysis and assessment were carried out on the PDMs. We estimated the residual PDMs and analyzed the polarimetric accuracy. Both the methods based on reflection symmetry and scattering reciprocity were performed. The polarization distortion errors of the GF-3 standard images are shown in Table 3. The crosstalk errors estimated by the two methods are different: the reflection symmetry method overestimates it (marked as Sym. in table), while the scattering reciprocity method underestimates it (marked as Rec. in table). Hence, we list both of the results separately. The channel imbalance errors calculated by these two methods are almost the same, so we show the average results. The co-imba errors were computed by CRs; therefore, there are only five images with these results. Generally speaking, the PDMs vary with the slant range, i.e., the incidence angle of the image, so we give here the mean value and standard deviation, for simplicity. The residual errors that exceed the accuracy requirement of the GF-3 satellite (crosstalk −35 dB, channel imbalance 0.5 dB, phase ±10 degrees) are marked in blue.

The results in Table 3 show that the channel isolation in GF-3 is controlled very well, and the crosstalk results of both methods satisfy the accuracy requirements. However, for the channel imbalance parameter, there are two values in one image exceeding the requirement slightly, and one value in one image is obviously abnormal. On the other hand, the system noise level of GF-3 is very low, in that the SNRs of all the images are better than 18 dB. In this situation, the noise has no influence on the PDM estimation and will not decrease the image quality [29].

We took the PolCAL processing of the standard GF-3 images and then re-estimated the residual errors to verify the effectiveness of the PolCAL method. We selected the reflection symmetry method for the PolCAL of the images, for its robustness. Furthermore, we estimated the residual errors using both of the methods. The results are shown in Table 4. We can see that, after PolCAL, the residual errors are substantially reduced for most of the cases. In addition, the crosstalk parameters that were estimated by the scattering reciprocity method at the Wuhan site are quite low. This because this method will underestimate the crosstalk, as described above, and the most objective way to evaluate image quality is using PARCs totally. On the other hand, these results are the best achievable residuals because they are obtained by calibrating each image with the PDMs computed from the image itself. However, two error values in image 0002441641 became larger than before (marked in red). This exception is a result of the experiment design. Since the polarimetric distortions are quite different between standard GF-3 image 0002441640 and image 0002441641, the deviation is generated when we calibrate image 0002441641 with the PDMs estimated by image 0002441640. This situation will be avoided when we calibrate image with PDMs estimated by itself, as shown at the Wuhan site, i.e., the last four images. Apart from this, the crosstalk is below −42 dB and the channel imbalance is better than 0.26 dB and ±0.2 degrees. This indicates that the PolCAL is effective, and it can clearly improve the image polarimetric quality.

## 5. Conclusions

The GF-3 satellite is the first multi-polarization SAR imaging satellite in China for civil use, and it has three full-polarization imaging modes, for which the image polarimetric quality is vital for the increasing PolSAR research group and applications. In this study, we assessed the image polarimetric quality and carried out a PolCAL experiment to improve the accuracy. PolCAL methods based on both reflection symmetry and scattering reciprocity were analyzed and utilized. The experimental results show that the polarimetric accuracy of these GF-3 images is high, and it can meet the accuracy requirements practically. The experiments also indicate that the PolCAL processing of the standard GF-3 images can clearly improve the image polarimetric quality, with crosstalk of −42 dB and channel imbalance of 0.26 dB in amplitude and ±0.2 degrees in phase.

## Figures and Tables

**Table 1:** Imaging modes and parameters of the GF-3 satellite.

Imaging Mode		Incidence Angle (◦)	Look Number	Resolution (m)	Imaging Width (km)	Polarization Mode
**Spotlight**		20~50	1 × 1	1	10	Selective single-polarization
**Ultra-fine strip**		20~50	1 × 1	3	30	Selective single-polarization
**Fine strip I**		19~50	1 × 1	5	50	Selective dual-polarization
**Fine strip II**		19~50	1 × 2	10	100	Selective dual-polarization
**Standard strip**		17~50	3 × 2	25	130	Selective dual-polarization
**Narrow scan**		17~50	1 × 6	50	300	Selective dual-polarization
**Wide scan**		17~50	1 × 8	100	500	Selective dual-polarization
**global**		17~53	2 × (2~4)	500	650	Selective dual-polarization
**Full-polarimetric strip I**		20~41	1 × 1	8	30	full-polarization
**Full-polarimetric strip II**		20~38	3 × 2	25	40	full-polarization
**Wave imaging**		20~41	1 × 2	10	5	full-polarization
**Extend**	**Incidence angle I**	10~20	3 × 2	25	130	Selective dual-polarization
	**Incidence angle II**	50~60	3 × 2	25	80	Selective dual-polarization

**Table 2:** Specific parameters of the experimental GF-3 images.

Imaging Region	Acquisition Date	Image ID	Central Look Angle	Image Size (Pixels)
**Etuoke Banner**	12 June 2017	0002441640	39.98	5881*4717
		0002441641	39.97	5778*4717
	24 June 2017	0002441191	43.92	7001*6161
		0002441190	43.91	7054*6161
	06 July 2017	0002463755	47.45	3775*5977
		0002463754	47.44	3814*5977
	11 July 2017	0002475549	40.01	6165*4708
		0002475550	40.01	6206*4708
	16 July 2017	0002491702	30.43	6770*7182
		0002491701	30.43	6778*7182
**Wuhan**	30 April 2017	0002335427	36.21	7982*6200
	30 April 2017	0002335421	36.22	7944*6200
	24 August 2017	0002559955	36.15	7464*6209
	02 October 2017	0002645747	37.54	5821*5959

**Table 3:** Polarization distortion errors of the standard GF-3 images.

Image ID	Crosstalk-Sym. (dB)	Crosstalk-Rec. (dB)	|α| (dB)	Arg(α) (deg.)	|k| (dB)	Arg(k) (deg.)	SNR (dB)
**0002441640**	−40±4.4	−48±3.4	−0.35±0.10	3.6±0.3	0.45	−6.5	19
**0002441641**	−45±3.4	−51±1.9	−0.20±0.05	3.2±0.2	−	−	19
**0002441191**	−37±4.0	−46±4.4	−0.28±0.15	0.8±0.3	−0.10	−5.4	18
**0002441190**	−36±2.9	−46±2.5	−0.22±0.04	0.8±0.2	−	−	19
**0002463755**	−40±3.8	−48±3.4	0.55±0.19	2.6±0.1	0.57	−2.3	20
**0002463754**	−38±2.4	−49±1.1	0.48±0.01	2.7±0.1	−	−	19
**0002475549**	−39±4.3	−48±3.8	−0.43±0.07	−1.6±0.4	−0.16	−7.4	18
**0002475550**	−41±3.3	−49±1.8	−0.30±0.03	−1.6±0.2	−	−	19
**0002491702**	−43±4.1	−47±2.7	−0.16±0.06	3.5±0.4	0.31	−9.5	21
**0002491701**	−46±3.9	−47±1.3	−0.18±0.03	3.6±0.1	−	−	20
**0002335427**	−35±8.8	−47±2.8	−0.44±0.05	−6.3±0.6	−	−	18
**0002335421**	−50±7.1	−61±1.5	1.04±0.02	−6.5±0.5	−	−	19
**0002559955**	−37±7.6	−51±4.2	−0.39±0.04	3.4±0.5	−	−	19
**0002645747**	−42±6.2	−59±5.3	−0.39±0.01	7.6±0.1	−	−	20

**Table 4:** Polarization residual errors after PolCAL.

Image ID	Crosstalk-Sym. (dB)	Crosstalk-Rec. (dB)	|α| (dB)	Arg(α) (deg.)	|k| (dB)	Arg(k) (deg.)	SNR (dB)
**0002441640**	−50±6.8	−66±10.5	0.07±0.07	−0.1±0.3	−0.05	−0.1	19
**0002441641**	−40±3.1	−54±3.1	0.25±0.07	−0.3±0.4	−	−	19
**0002441191**	−50±6.8	−64±9.8	0.10±0.10	0.1±0.3	−0.05	0.01	18
**0002441190**	−42±5.4	−62±7.3	0.09±0.08	−0.03±0.2	−	−	19
**0002463755**	−52±6.2	−69±9.5	−0.10±0.14	−0.01±0.1	0.06	−0.01	20
**0002463754**	−45±5.0	-66±7.6	−0.14±0.03	0.1±0.1	−	−	19
**0002475549**	−47±7.8	−65±12.5	0.10±0.04	0.04±0.3	−0.06	0.01	18
**0002475550**	−46±4.6	−60±7.2	0.26±0.06	0.1±0.3	−	−	19
**0002491702**	−58±6.8	−69±10.3	0.04±0.05	0.04±0.4	−0.02	0.01	21
**0002491701**	−51±6.7	−66±5.9	0.02±0.04	0.2±0.1	−	−	20
**0002335427**	−45±7.2	−78±4.6	0.07±0.01	0.01±0.01	−	−	18
**0002335421**	−67±6.4	−98±5.7	−0.14±0.01	−0.01±0.01	−	−	19
**0002559955**	−46±8.4	−81±4.7	0.07±0.01	0.01±0.01	−	−	19
**0002645747**	−48±6.4	−90±5.8	0.05±0.01	0.01±0.01	−	−	20
